# Correction to: Tempo-dependent selective enhancement of neural responses at the beat frequency can be mimicked by both an oscillator and an evoked model

**DOI:** 10.1093/cercor/bhag039

**Published:** 2026-03-20

This is a correction to: Atser Damsma, Mitchell de Roo, Keith Doelling, Pierre-Louis Bazin, Fleur L Bouwer, Tempo-dependent selective enhancement of neural responses at the beat frequency can be mimicked by both an oscillator and an evoked model, *Cerebral Cortex*, Volume 35, Issue 9, September 2025, https://doi.org/10.1093/cercor/bhaf258

The following changes have been made to the originally published paper. The statement of the **Funding** section has been changed to read: “A.D. was supported by an Amsterdam Brain and Cognition (ABC) Project Grant awarded to Henkjan Honing and Pierre-Louis Bazin. F.L.B. was supported by a VENI grant from the Dutch Research Council NWO (VI.Veni.201G.066).” instead of: “This work was supported by an Amsterdam Brain and Cognition (ABC) Project Grant to A.D. and a VENI grant from the Dutch Research Council NWO (VI.Veni.201G.066) to F.L.B.”

An **Acknowledgement** section has been added to the paper to read: “We thank Henkjan Honing, supporting applicant of the ABC Project Grant, for his role in funding acquisition and for providing the institutional support to host the project within the Music Cognition Group at the Institute for Logic, Language, and Computation (ILLC).”

